# Understanding speech and language in tuberous sclerosis complex

**DOI:** 10.3389/fnhum.2023.1149071

**Published:** 2023-06-01

**Authors:** Tanjala T. Gipson, D. Kimbrough Oller, Daniel S. Messinger, Lynn K. Perry

**Affiliations:** ^1^Department of Pediatrics, Le Bonheur Children’s Hospital, The Boling Center for Developmental Disabilities, University of Tennessee Health Science Center, Memphis, TN, United States; ^2^School of Communication Sciences and Disorders, Institute for Intelligent Systems, The University of Memphis, Memphis, TN, United States; ^3^Department of Psychology, University of Miami, Miami, FL, United States; ^4^Department of Pediatrics, University of Miami, Miami, FL, United States; ^5^Department of Electrical and Computer Engineering, University of Miami, Miami, FL, United States; ^6^Department of Music Engineering, University of Miami, Miami, FL, United States

**Keywords:** autism spectrum disorder, tuberous sclerosis complex (TSC), language, speech, neurodevelopmental disorders

## Abstract

Tuberous Sclerosis Complex (TSC), is a neurocutaneous disorder, associated with a high prevalence of autism spectrum disorder (ASD; ∼50% of individuals). As TSC is a leading cause of syndromic ASD, understanding language development in this population would not only be important for individuals with TSC but may also have implications for those with other causes of syndromic and idiopathic ASD. In this mini review, we consider what is known about language development in this population and how speech and language in TSC are related to ASD. Although up to 70% of individuals with TSC report language difficulties, much of the limited research to date on language in TSC has been based on summary scores from standardized assessments. Missing is a detailed understanding of the mechanisms driving speech and language in TSC and how they relate to ASD. Here, we review recent work suggesting that canonical babbling and volubility—two precursors of language development that predict the emergence of speech and are delayed in infants with idiopathic ASD—are also delayed in infants with TSC. We then look to the broader literature on language development to identify other early precursors of language development that tend to be delayed in children with autism as a guide for future research on speech and language in TSC. We argue that vocal turn-taking, shared attention, and fast mapping are three such skills that can provide important information about how speech and language develop in TSC and where potential delays come from. The overall goal of this line of research is to not only illuminate the trajectory of language in TSC with and without ASD, but to ultimately find strategies for earlier recognition and treatment of the pervasive language difficulties in this population.

## 1. Introduction

Autism spectrum disorder (ASD) can be classified as syndromic or idiopathic based on whether the cause is known or unknown, respectively (Lord et al., 1997). Tuberous sclerosis complex (TSC), a genetic, neurocutaneous condition, is a leading cause of syndromic ASD accounting for approximately 1–3% of such cases (Smalley et al., 1992). Additionally, nearly half of those with TSC have ASD (de Vries et al., 2007). Further, up to 70% of individuals with TSC also have language difficulties regardless of the presence of ASD (de Vries and Bolton, 2002). However, little is known about the mechanisms of speech and language development in this population, whether they differ from those driving development in typically developing children, and how they might relate to the TSC and ASD-specific symptoms. Longitudinal research capturing more fine-grained changes in speech and language development and its relationship to ASD in TSC is needed and may aid in our understanding of language in ASD more generally. In this mini review, we consider the literature to date about speech and language in TSC and look to the literature on speech and language in ASD more broadly as a guide toward future research on TSC.

### 1.1. Epidemiology and clinical features of TSC

Tuberous Sclerosis Complex occurs in 1/6000 live births impacting up to 1 million individuals worldwide (Osborne et al., 1991; O’Callaghan, 1999; Curatolo et al., 2008; Hallett et al., 2011; Hong et al., 2016). It is classified as a neurocutaneous disorder due to the presence of non-cancerous brain lesions and skin manifestations (Northrup et al., 2021). A clinical diagnosis of TSC is established in the presence of at least 2 major or 1 major and 2 minor features (Table 1) or genetically by identification of a mutation in *TSC1* or *TSC2*. Seizures impact 90% of the population and often start in infancy (Nabbout et al., 2019). ASD and ASD-like features are among the known neuropsychiatric comorbidities of TSC collectively termed TAND (TSC-associated neuropsychiatric disorders) (de Vries et al., 2015, 2018a,b; Leclezio et al., 2018). Phenotypically, social communication deficits in children with TSC-associated autism are similar to the social communication deficits of children with ASD without TSC according to results from the ADOS (Jeste et al., 2008, 2016), and these types of deficits were significantly more common than restrictive, repetitive behaviors (Moavero et al., 2019). However, ASD-like symptoms were found even in participants with TSC who did not have ASD (Capal et al., 2021).

**TABLE 1:** Major and minor features of tuberous sclerosis complex.

Major features	Minor features
Subependymal nodules (SEN)	Multiple renal cysts
Subependymal giant cell astrocytomas (SEGA)	Non-renal hamartomas
Cortical dysplasias*	Dental pits (> 3)
Renal angiomyolipomas	Intraoral fibromas (≥ 2)
Pulmonary lymphangioleiomyomatosis (LAM)	“Confetti” skin lesions
Cardiac rhabdomyomas	Retinal achromic patch
Angiofibromas (≥ 3)	
Hypopigmented macules (≥ 3, at least 5-mm)	
Cephalic plaque	
Ungual fibromas (≥ 2)	
Shagreen patch	
Multiple retinal hamartomas	

Clinically definite TSC is defined as having 2 or more major features or 1 major and ≥2 minor features. Clinically possible TSC is defined as having 1 major or ≥2 minor features. LAM and renal angiomyolipomas in combination are classified as 1 major feature of TSC.

*Cortical dysplasias include cortical tubers and white matter radial migration lines.

## 2. Language and speech abilities in TSC

### 2.1. Impact of neurobiology on language and development in TSC

The underlying cause of both ASD and language difficulties in TSC is unclear. However, a small but growing body of research is beginning to assess speech and language abilities in children and adults with TSC and the potential association between these abilities and ASD symptoms. For example, one recent large-scale study found that about half of the participating individuals with TSC/their caregivers of children with TSC reported they/their child had language delays on the TAND Checklist, a validated interview used to screen for TSC-associated neuropsychiatric disorders (TAND). However, the prevalence of these language delays varied with IQ: 27.8% of individuals with normal IQ reported language delays, 62.4% of those with mild-moderate intellectual disability and 88.2% of those with severe/profound intellectual disability (Alperin et al., 2021). Another recent large-scale study of TSC followed participants longitudinally from infancy, reporting secondary outcomes of TSC including language abilities using developmental quotient scores from the Bayley Scales of Infant Development (BSID-III). The number of children scoring in the normal ranges for their age, decreased over development. At 6 months, 70% of the cohort’s BSID scores fell within the typical range for typically developing children, but by 2 years only 45% of the scores were in the typical range. Although it is unclear whether these declines were due to regression or failure to progress, a progressive decline in the language developmental quotient was associated with an increased likelihood of receiving an ASD diagnosis by 2 years (Moavero et al., 2019).

Language delays and ASD in children with TSC are likely the product of the TSC-specific brain lesions and differences in functional connectivity between regions with relevance to these outcomes. For example, magnetoencephalography of adults with and without TSC without intellectual disability during a semantic decision task, revealed differences in the functional connections between Broca’s and Wernicke’s, brain regions involved in language use and processing (Gallagher et al., 2013). In those with TSC, activation in Broca’s and Wernicke’ occurred nearly simultaneously. In adults without TSC, however, this activation occurred serially (Gallagher et al., 2013). Simultaneous activation of Broca’s and Wernicke’s areas may lead to difficulties with language processing because there is no time lapse between the signal for comprehension (Wernicke’s) and spoken language (Broca’s). Underconnectivity has also been found in children with TSC-associated ASD during the first 2 years of life in regions involved in speech and language, including the sagittal stratum (language processing), cingulum (emotion processing), anterior limbs of the internal capsule (speech), arcuate fasciculus (spontaneous speech, repetition, word retrieval and comprehension), and the corpus callosum (transfer and integration of language between the hemispheres) (Prohl et al., 2019).

Other neural differences in TSC are more directly related to ASD symptoms, which itself can have consequences for language development. For example, in a recent study, an increased number of cortical lesions on fetal brain MRI in children with TSC was linked to development of ASD by age 2 years (Hulshof et al., 2021). Decreased connectivity of the fusiform gyrus, which is responsible for face recognition and is a part of the “social brain network,” was associated with a greater risk of developing ASD in TSC (Scherrer et al., 2020).

Critically, these differences in functional connectivity can occur even in the absence of noticeable brain lesions in the regions, suggesting that other differences in TSC, such as seizures or differences in lived experiences as a consequence of frequent seizures interrupting typical social interactions, can play a role in the development of neural connections.

#### 2.1.1. Impact of epilepsy on language and development in TSC

The diagnosis of epilepsy, early onset seizures, and epilepsy severity indicated by more frequent seizures disrupt typical brain function, especially in the young. Seizures can have deleterious effects on language development (Croft et al., 2014). However, the exact mechanisms driving these effects are unclear. Indeed, pharmaceutical intervention studies suggest the timing of first seizure does not itself lead to differences in language or ASD diagnosis. For example, although children who received vigabatrin, an antiseizure medication, before seizures started experienced a reduced risk of clinical seizures, drug resistant epilepsy, and infantile spasms than those who received vigabatrin after seizures started (Kotulska et al., 2021), there were no differences in these two groups’ language abilities or likelihood of being diagnosed with ASD or developmental delay by age 2 years (Moavero et al., 2020).

Epilepsy severity, or more frequent seizures, is also associated with language development. The number of antiseizure medications children took (an index of epilepsy severity) was associated with their expressive vocabulary size at 8–36 months, and with their receptive vocabulary size and gestures at 8–18 months, such that children taking more medications had smaller vocabularies (Forys-Basiejko et al., 2022). Differences in expressive vocabulary were also associated with duration of seizures and response to treatment with antiseizure medications, such that children with larger expressive vocabularies tended to have shorter seizure duration (less than 6 months) and required fewer antiseizure medications (Forys-Basiejko et al., 2022). Epilepsy is known to cause disruptions in development, and the studies reviewed here support its role in disrupting typical language development in those with TSC.

## 3. Characterizing speech, language, and ASD in TSC

Early language abilities in TSC have been associated with later ASD symptoms and diagnosis. Receptive and expressive language abilities of 6-month-old infants with TSC, as measured by the BSID III, are negatively associated with their likelihood of developmental delays at 24 months. Lower language ability scores on other standardized assessments, including, e.g., the Mullen Scales of Early Learning, the Vineland Adaptive Behavior Scales, and the Preschool Language Scales, are also associated with a later diagnosis of ASD (Schoenberger et al., 2020). Indeed, although features of epilepsy in TSC have been associated with later ASD diagnosis, language assessment scores are reportedly a better predictor of ASD than seizure onset (Capal et al., 2017), underscoring the need for better understanding of language development in children with TSC.

Notably, to date, the few studies that characterize speech and language in children with TSC have relied almost exclusively on caregiver reports or standardized assessments that only give a broad view of language abilities and fail to characterize specific aspects of speech and language or developmental mechanisms that might be affected by TSC symptoms or might most reliably predict ASD diagnosis. Detailed evaluation comparable to the kinds of research that occurs commonly in the general fields of child language development (Oller and Lynch, 1992; Oller et al., 2010; Gangi et al., 2014; Perry et al., 2016; Long et al., 2022) is essentially absent.

One exception is recent work by Gipson et al. (2021). In their study, two features of early prelinguistic vocalizations, canonical babbling and volubility, were assessed using audio-video recordings of infants with TSC from the TACERN database. Canonical babbling is the utterance of consonant vowel combinations, e.g., “baba” and “dada” with onset typically by age 6 months. The canonical babbling ratio (CBR) is the proportion of canonical syllables to the number of total syllables uttered. Volubility is the number of utterances produced per minute (Lynch et al., 1995; Oller et al., 1997). Canonical babbling and volubility are both necessary precursors of typical spoken language and have been shown to be disrupted in those with language disorders and/or ASD which (Patten et al., 2014; Belardi et al., 2017), but had not previously been studied in TSC. In most of the infants with TSC, entry into the canonical babbling stage was delayed, and the canonical babbling ratio was low compared to typically developing infants (Gipson et al., 2021). Further, volubility in infants with TSC was about half that of typically developing infants. Delays in these vocalization features might be the earliest sign of adverse outcomes in language and development in children with TSC, and if missed, could limit opportunities for early targeted intervention. In general, more research of this kind in children with TSC—multi-measure, longitudinal research with appropriate comparison groups, detailed measures of prelinguistic and linguistic behaviors, will be important for understanding the links between ASD and language development in this condition and more broadly. In the next section we highlight three example skills for such targeted examinations.

## 4. Language development skills for future research in TSC

There are a number of skills related to the interactive and domain general mechanisms necessary for learning language, but to date none of these have been studied in TSC. We describe three such skills, vocal turn-taking, shared attention, and fast-mapping, below. We identified these three skills out of the many children develop, due to the insight potential investigations could yield about TSC. First, all three skills have been shown to systematically differ in children with and without ASD. As about half of individuals with TSC receive an ASD diagnosis, gaining insight into the associations between these aspects of language and their association with ASD in TSC in particular will be valuable for early diagnosis and intervention of ASD symptoms. Second, all three skills tend to begin developing within the first few years of life in typically developing children. As TSC is a condition that can be recognized early (during the third trimester of pregnancy), and its primary features, brain lesions and seizures occur very early, we should expect early building blocks of language to be affected. Third, all three are mechanistic skills that help children learn *how* to learn language rather than specific bits of linguistic knowledge that children might or might not have. Thus, unlike knowing that a child’s score on an assessment suggests they have a delay, understanding how these skills might differ in children with TSC tells us something about *how and why* language might be delayed in TSC and gives us potential pathways for target interventions.

### 4.1. Vocal turn-taking

*Vocal turn-taking*, or back-and-forth vocal exchanges between individuals, is an important skill that develops pre-linguistically. When adults vocally respond to an infant’s vocalization, the infant learns both about the contingent conversational nature of language and about specific words and speech sounds (Warren et al., 2010). Indeed, previous research has shown that children who engage in more vocal turn-taking with their caregivers have faster rates of vocabulary development and better long-term language outcomes (Gilkerson et al., 2018). Vocal-turn-taking between a child and a caregiver has been characterized as a social feedback loop in which both the child and the caregiver are learning from and influencing each other’s behavior (Warlaumont et al., 2014). For example, caregivers are more likely to respond to children’s speech-like vocalizations than non-speech like vocalization. This feedback loop can be disrupted in the context of ASD. Because children with ASD tend to engage in less canonical babbling, producing fewer speech-like vocalizations, their caregivers respond less contingently to them, leading to lower rates of vocal turn-taking relative to children without ASD (Oller et al., 2010; Warlaumont et al., 2014). The delays in canonical babbling and volubility in infants with TSC (Gipson et al., 2021) could have similar cascading consequences on vocal turn-taking behavior., These delays may render caregivers less likely to respond to their vocalizations, decreasing their own likelihood of responding to the caregiver’s responses over time. In children who have TSC-associated ASD, this social feedback loop could be disrupted further.

### 4.2. Shared attention

Early delays in infant vocalizations and vocal turn taking likely have a cascading effect on other critical building blocks of language, including shared attention. *Shared attention* is two individuals both having a common focus on the same thing. In the context of caregiver-child interactions, often this shared attention involves both caregiver and child attending to the same object. Shared attention can help children learn the meaning of the words caregivers say, as it disambiguates the referent of a new word in the moment (Edmunds et al., 2017). The contingent responsive behaviors necessary for vocal turn taking likely also play a role in the development of shared attention. With repeated interaction, children learn that vocalizing, pointing, and eye gaze are cues to attention, and begin to follow them to understand others’ attention and use them to communicate their own attention. But if the social feedback loop is disrupted and children and caregivers do not engage in these sorts of interactions, there would be fewer opportunities for children to learn about these cues and others’ attention, delaying shared attention. Indeed, delays in shared attention are strongly predictive of ASD (Tomasello et al., 2005; Mundy, 2016). Furthermore, given the underconnectivity in the social brain network and general social communication deficits observed in TSC, it is likely that there could also be delays in shared attention skills.

### 4.3. Fast mapping

Finally, delays in early vocalizations, difficulty with vocal turn-taking and deficits in shared attention could lead to deficits in the ability to learn and remember new words in children with TSC. *Fast mapping*, the ability to learn the association between a new word and its referent after a brief exposure (Horst and Samuelson, 2008), is one way children learn new words. As a group, typically developing 24-month-olds can successfully retain new words if their referents are explicitly named (Horst and Samuelson, 2008; Perry et al., 2016), and this skill has been linked to both concurrent vocabulary (Perry et al., 2016; Samuelson et al., 2017) and later vocabulary knowledge (Bion et al., 2013). The associations between fast mapping and vocabulary suggest that the process of learning new words teaches children *how* to learn new words. As they learn words, they become better word learners, more likely to remember associations between words and referents after a brief exposure. However, children with ASD tend to show differences or delays in their fast mapping skills compared to children without ASD, potentially due to vocabulary delays (Venker et al., 2016; Hartley et al., 2019; Joseph et al., 2019). Although fast mapping has not been studied in TSC, the vocabulary delays shown in children with TSC, especially those with epilepsy and ASD, lead us to hypothesize that there could be similar delays in fast mapping. Epilepsy may also directly affect language learning in this population due to frequent hospitalizations, the phase of recovery following the seizure, and the excessive sleepiness caused by antiseizure medications and limiting opportunities for social interactions and word learning. Importantly, understanding the fast mapping process in this population may have treatment implications, such as provision of social feedback, which seems to improve retention and generalization following fast mapping, particularly for children with ASD (Hartley et al., 2019).

## 5. Conclusion

Better understanding of these and other aspects of language and speech development in individuals with TSC with and without ASD is important as it can lead to more targeted interventions and inform our approach to understanding and treating speech and language difficulties in other forms of syndromic and idiopathic ASD.

## Author contributions

TG wrote the original draft, reviewed and edited the revisions, and reviewed/approved the final manuscript. DO and LP reviewed and edited the revisions and reviewed/approved the final manuscript. DM reviewed/approved the final manuscript. All authors contributed to the article and approved the submitted version.
